# Supplementary material for: Modulator Effect of AT1 Receptor Knockdown on THP-1 Macrophage Proinflammatory Activity
Source: Biology (Basel). 2024 May 26;13(6):382. doi: 10.3390/biology13060382 (PMC11200961; doi:10.3390/biology13060382)
Supplement: Supplementary file 1 [file biology-13-00382-s001.zip › biology-2987814-supplementary.pdf]

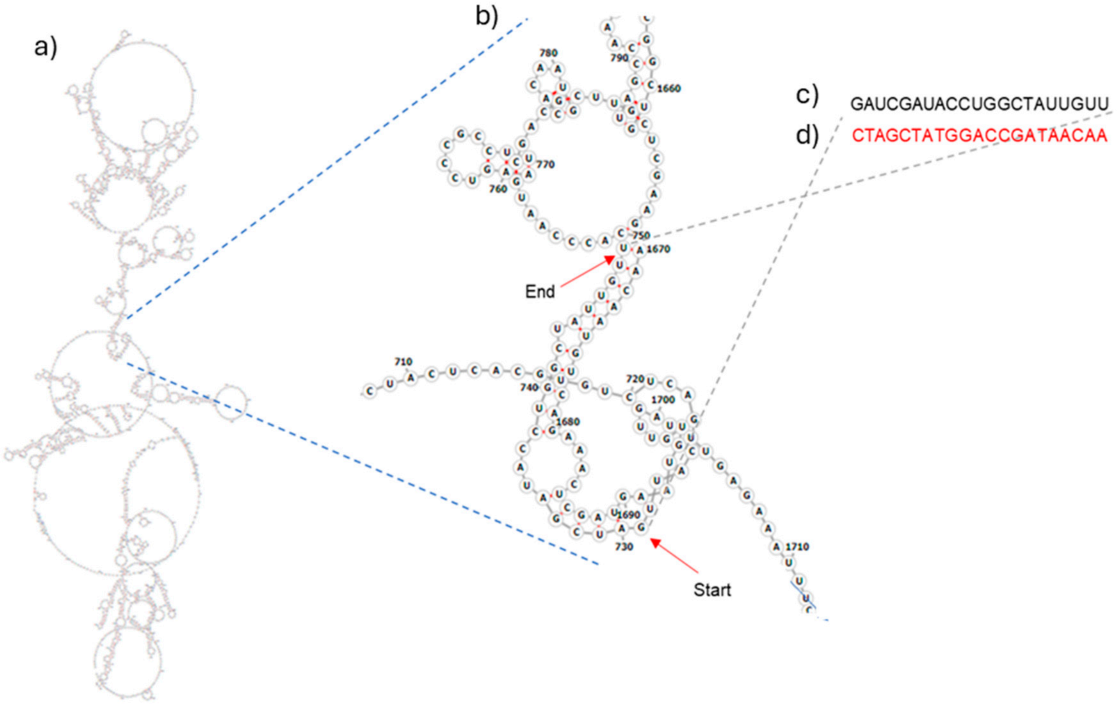

Figure S1. Site of binding of siRNA to messenger RNA of AT1. a) Secondary structure of mRNA AT1, b) mRNA AT1 zoom, c) hybridization site sequence, d) siRNA AT1.
